# Plasticity in neurological disorders and challenges for noninvasive brain stimulation (NBS)

**DOI:** 10.1186/1743-0003-6-4

**Published:** 2009-02-17

**Authors:** Gary W Thickbroom, Frank L Mastaglia

**Affiliations:** 1 Centre for Neuromuscular and Neurological Disorders, University of Western Australia, Nedlands, Western Australia, Australia

## Abstract

There has been considerable interest in trialing NBS in a range of neurological conditions, and in parallel the range of NBS techniques available continues to expand. Underpinning this is the idea that NBS modulates neuroplasticity and that plasticity is an important contributor to functional recovery after brain injury and to the pathophysiology of neurological disorders. However while the evidence for neuroplasticity and its varied mechanisms is strong, the relationship to functional outcome is less clear and the clinical indications remain to be determined. To be maximally effective, the application of NBS techniques will need to be refined to take into account the diversity of neurological symptoms, the fundamental differences between acute, longstanding and chronic progressive disease processes, and the differential part played by functional and dysfunctional plasticity in diseases of the brain and spinal cord.

## Introduction

While there are a number of noninvasive brain stimulation (NBS) techniques that can alter indices of brain excitability, a lasting functional benefit from these interventions in clinical populations remains elusive. Initially driven by psychiatric applications, and modeled on the effectiveness of electro-convulsive therapy (ECT), there is increasing interest in how neuromodulation by noninvasive brain stimulation (NBS) might be extended to neurological disorders. Underpinning this is the idea that NBS modulates neuroplasticity and that plasticity is important in the pathophysiology of neurological disorders and plays an important role in functional recovery and adaptation to neurological deficits. However while the evidence for neuroplasticity and its underlying mechanisms is strong, the relationship to functional outcome is less clear and somewhat theoretical. A re-appraisal of the contribution of brain plasticity to the symptomatology and functional outcome in neurological disorders may help guide the clinical application of NBS, and is the topic of this review.

### What is brain plasticity?

The term plasticity as applied to the brain usually refers to adaptability and reorganization, rather than large-scale malleability (i.e. to 'software' rather than 'hardware' modifications). However in keeping with the original design principle of plasticine, namely that it would not harden (invented near Bath in 1897 by William Harbutt, early samples have remained plastic for ~100 years), there is no age limit in principle to the brain's adaptability or ability to undergo plastic changes, only the degree and form vary [1].

Brain plasticity may be neuronal or non-neuronal [e.g. astrocyte-mediated; [2]], and neuronal plasticity in turn may be synaptic or non-synaptic [e.g. changes in intrinsic excitability; [3]]. Given the fundamental importance of synaptic transmission to brain function, it is the synapse that incorporates the greatest range of mechanisms of action and potential for plasticity (e.g. pre- and post-synaptic, molecular and ionic, neurotransmitter dynamics, receptor function and structure, retrograde messengers, dendritic signaling; [see [4]]).

Synaptic plasticity may be further characterized according to its spatial scale and mode of induction. Plasticity on an intra-network scale can be thought of as a relatively-localized change in synaptic weighting (or fine-scale synaptic sprouting) within a functional neuronal unit such as a neocortical column. Inter-network plasticity can be thought of as a larger-scale remodeling (within or between cerebral hemispheres) in the pattern of activity in a network that serves a given brain function such as the motor network, or even across functional networks, such as recruitment of visual cortex during Braille reading in the blind [5] or activation of auditory cortex during visual stimulation in the deaf [6]. The most apparent clinical manifestation is the increased activation reported in the non-lesioned hemisphere after unilateral stroke [7]. These forms of plasticity do not represent large-scale structural changes in connectivity as the ability to repair damage to white matter tracts in the mature brain is severely limited.

The triggers and mechanisms for forms of synaptic plasticity will differ, but ultimately will depend on achieving a desired functional outcome such as consolidating a memory-trace, learning a new skill or compensating for brain damage. Two main principles of action have been identified, activity and time-dependent forms of plasticity [8]. A persistent increase in neuronal firing during task performance implies that the network involved has a functionally-significant role, and is one trigger for neuronal plasticity. Likewise, a precisely-timed relationship between neuronal activation within a network (cause-effect principle) implies that these neurons are cooperating in a functional way and are candidates for plasticity-related upregulation. Both of these forms of plasticity seem accessible to NBS techniques [9].

### Experimental basis for plasticity

The first experimental description of persisting changes in synaptic efficacy following neural stimulation was published in 1973 and described as long-term potentiation (LTP) of excitatory glutamatergic synapses [10]. Demonstration of long-term depression (LTD) at these synapses was later reported [11]. The favored model for these effects is through ionotropic N-methyl-D-aspartic acid (NMDA) mediated modulation of the number and conductance of AMPA (alpha-amino-3-hydroxy-5-methyl-4-isoxazolepropionic acid) receptors [see [12,13]]. Other mechanisms have since been implicated in plasticity of glutamatergic synapses, particularly those mediated by metabotropic G-protein-coupled receptors (mGluRs) [14].

More recently, LTP and LTD of inhibitory GABAergic synapses have been described [15], and as is the case for glutamatergic synapses, both ionotropic and metabotropic mechanisms are involved. The presence of plasticity mechanisms across multiple forms of neurotransmission is needed to retain overall balance (for example to retain temporal fidelity mediated by inhibitory synapses in the presence of increased excitability of glutamatergic synapses [16]). As well, mechanisms for regulating plasticity (homeostasis and metaplasticity) are needed to keep the system at a balance point [17,18]. Many other neurotransmitters contribute to plasticity or its regulation, for example dopamine [19]. Together, they give the brain a battery of mechanisms with which to respond to injury or to adapt to changing circumstance, but as with any profoundly complex system, a breakdown in any component can lead to significant consequences. Thus plasticity can be regarded as functional or dysfunctional, and this distinction is likely to be important for the application of NBS in clinical situations.

### Plasticity in neurology

To be effective, NBS interventions must take into account the range of neurological disorders, their heterogeneity even within well-defined and characterized conditions, and the diverse time courses over which they act, from acute self-limiting injuries such as stroke, through chronic progressive disorders such as Parkinson's disease, to more established and persistent conditions such as dystonia.

#### Stroke

This model may present the most promise for the application of NBS if the intervention can facilitate a longer-lasting recovery in the absence of further brain damage. The contribution of neural plasticity in recovery from stroke is suggested by changes in cortical maps identified by transcranial magnetic stimulation (TMS) and changes in activation patterns observed with functional imaging [20-22]. In the case of TMS mapping, a correlation has been reported between grip-strength in the affected hand and the extent of cortical map shifts, suggesting this form of cortical plasticity may be beneficial to function [22]. There has been some modest functional improvement reported after some NBS interventions, however the longer-term clinical benefits remain unproven [23] and it is likely that NBS will need to be administered in combination with other therapies for more lasting effects; however the relative timing and the nature of the intervention and the therapy remains to be determined, and some combinations may be detrimental. It seems certain that the direct application of a non-specific NBS intervention in stroke is unlikely to be successful. Other acute disorders include traumatic brain and spinal cord injury and inflammatory diseases such as multiple sclerosis. In each of these conditions it is likely that plasticity is functional rather than dysfunctional and may contribute to an improvement in symptoms. However, plasticity could also contribute to dysfunction such as spasticity after stroke or brain injury early in life (e.g. cerebral palsy).

#### Parkinson's disease

Chronic progressive diseases are a challenge for NBS. The evolution of these diseases occurs over the longer-term and is constantly changing, whereas NBS is difficult to administer chronically and probably does not have the flexibility to manage a constantly changing baseline. Parkinson's disease (PD) is a progressively developing movement disorder arising from loss of dopaminergic neurons in the substantia nigra and depletion of dopamine in the basal ganglia. Although the pathology is subcortical, secondary abnormalities manifest in cortical structures, including changes in cortical inhibition and shifts in the cortical representation of hand muscles which can occur in both early and late stages of the disease [24,25]. Map shifts correlate with the severity of clinical symptoms (UPDRS) and suggest an ongoing process of cortical reorganization with functional consequences [24]. Dopamine has been implicated in the modulation of neuroplasticity [19], and the loss of dopaminergic neurons in PD may have secondary effects on cortical organization or limit the natural ability of plasticity mechanisms to compensate for disease-related processes, and there is some indication that NBS may be more effective when applied during levodopa therapy, when plasticity mechanisms may be more functional [26,27]. As well, cortical rTMS interventions can lead to release of dopamine in the basal ganglia and raise serum dopamine levels [28]. As to whether NBS can have a lasting benefit in a progressive disease such as PD, in which the primary pathology is subcortical, and which manifests as a generalized disorder, is uncertain. However a number of NBS interventions have been trialed in PD and have yielded some modest if transient functional improvement, and meta-analysis of randomized controlled trials in PD indicate NBS can be beneficial over and above placebo effects [29]. Plasticity in PD may be functional in the earlier stages of the disease, as the brain adapts to the initial loss of dopaminergic neurons, but is probably dysfunctional later in the progression of the disease as plasticity mechanisms become gradually impaired as a result of dopamine depletion.

#### Dystonia

Dystonia results from unwanted contraction of muscles that may be focal, generalized or task-specific and is thought to arise from alterations in basal ganglion regulatory loops involving premotor cortical areas and motor cortex [30,31]. There is evidence that plasticity is up-regulated in dystonia and probably dysfunctional. Changes in corticomotor excitability with NBS interventions are of greater magnitude, less spatially restricted and longer-lasting [31]. As well, an abnormality in metaplasticity has been inferred from the influence of a conditioning NBS intervention on the subsequent induction of plasticity [32]. TMS mapping studies reveal changes in the cortical representation of muscles that are primarily involved in the dystonic posture, as well as muscles that are not dystonic [33-35]. Alleviation of symptoms following injection of botulinum toxin into affected muscles is associated with normalization of TMS maps, suggesting that reorganisation is an ongoing and dynamic process perhaps maintained by abnormal afferent inputs to cortical regions [34,35]. How NBS could be applied therapeutically in dystonia is uncertain, although alleviation of symptoms has been reported with an excitability-reducing NBS protocol delivered over a fMRI-identified region of hyperactivity within dorsolateral prefrontal cortex [36]. In principle, any intervention to upregulate plasticity is probably contra-indicated. It is possible that interventions targeting metaplasticity may be able to change the set-point for the probability of inducing plasticity, and be a more promising approach. Interventions will also need to take into account the considerable heterogeneity in dystonia, from symptoms arising from discrete lesions in the basal ganglia or after trauma, to genetic and idiopathic forms and over-use syndromes.

### NBS techniques in neurology

There has been no lack of interest in trialing NBS in a substantial range of neurological conditions, and in parallel the range of NBS techniques available continues to expand. One of the first clinical applications in the modern era entailed delivering a train of low-frequency TMS to motor cortex in PD [37]. Experimental models suggest that these frequency-dependent stimulation protocols are probably up- or down-regulating the activity of excitatory glutamatergic synapses, and it follows therefore that these interventions are most suited to clinical situations such as Parkinson's disease in which thalamocortical activation is impaired and modulation of excitatory synaptic transmission is indicated. In general however, it is more likely to be the balance between excitation and inhibition that is impaired, and modulation of intracortical inhibition is likely to be only a secondary outcome of these interventions rather than the target. To date no effective TMS intervention is available to modulate the excitability of inhibitory GABAergic neurones, which would be applicable in hyperexcitability states such as epilepsy, although paired-pulse approaches have been trialed with some degree of success [38-40]. A reduction in seizure frequency has been reported in epilepsy after low-frequency rTMS [41], perhaps through LTD of glutamatergic transmission, and NBS appears relatively safe in epilepsy [42].

Frequency-dependent NBS is a form of activity-dependent plasticity. Other NBS models that have activity-dependent characteristics are theta-burst stimulation [TBS; [43]] and upregulation of activity with paired-associative stimulation [PAS; [44]]. Time-dependent plasticity is another form of plasticity that may be more physiological during functional learning when network activity must be coordinated to lead to meaningful function. Using paired pulse TMS at intervals corresponding to transynaptic transmission it is possible to emulate this more physiologically refined form of plasticity [45]. The nature of the neurological disorder will need to be considered when selecting between activity- and time-dependent interventions. Gross changes in overall excitability might suit activity-dependent models (e.g. in dystonia) whereas a time-dependent NBS model might be more appropriate with learning-related protocols as during stroke rehabilitation. In a different class altogether is transcranial DC stimulation, which is thought to target membrane excitability and secondarily NMDA receptor mechanisms. The possibility of modulating membrane excitability is novel and early results seem to indicate that this is a promising intervention across a range of neurological disorders and warrants further investigation [46,47]. Other newer NBS approaches continue to be developed and increase the range of potential applications in neurology [48].

## Summary

Unfortunately there is still much that is not known about the basis of many neurological conditions, and this makes it difficult to be certain as to which NBS interventions may be most suited to any given situation, but an awareness of these issues is important for deciding on the approach to use and for the further development of NBS protocols. Compounding this is the diversity of disorders themselves. Even with stroke, arguably the most suited to NBS therapy, brain damage can occur anywhere within the brain including subcortical structures, white matter tracts, cerebral cortex and underlying white matter, cerebellum, brainstem etc, and be of variable spatial extent and severity. Thus there is no such thing as 'a' stroke, and NBS interventions will need to accommodate this diversity. Finally, NBS interventions must take into account that plasticity in neurological disorders ranges from the functionally-beneficial to dysfunctional and detrimental, and therefore be sure that an intervention does not exacerbate dysfunctional plasticity. To be most effective, NBS techniques will need to be refined to incorporate the diversity of neurological symptoms and their temporal profiles and the different types of spontaneous neuroplasticity occurring in neurological disorders.

## Competing interests

The authors declare that they have no competing interests.

## Authors' contributions

GWT drafted the manuscript. FLM revised the manuscript. Both authors contributed to the plan of the manuscript, and read and approved the manuscript.
